# A Cadaveric Case Report of an Incomplete Double Ureter Associated With Testicular Arterial Variations

**DOI:** 10.7759/cureus.67613

**Published:** 2024-08-23

**Authors:** Peter W Deucher, Tori N Thorkildsen, Devin Farrell, Amer A Khan, Vanessa C Cornelio, Kamal A Abouzaid, Ahmad Imam

**Affiliations:** 1 Medicine, William Carey University College of Osteopathic Medicine, Hattiesburg, USA; 2 Anatomical Sciences, William Carey University College of Osteopathic Medicine, Hattiesburg, MS, USA; 3 Anatomical Sciences, William Carey University College of Osteopathic Medicine, Hattiesburg, USA

**Keywords:** bifid ureter, double ureter anomaly, arched testicular artery, accessory testicular artery, testicular artery anomaly, congenital anomalies of kidney, cadaver case report, nutcracker syndrome (ncs), congenital anomalies of the kidney and urinary tract (cakut), incomplete double ureter

## Abstract

This case report details anatomical variations in a cadaveric donor during the dissection laboratory. This case shows a possible association between an incomplete double ureter, arching testicular arteries, and an accessory testicular artery. This case describes these variations and briefly discusses ways to classify them. We aim to document these anomalies, discuss possible embryological reasons for their association, and shed light on their clinical significance. This case report contributes to the limited literature and highlights the importance of reporting these anomalies when encountered during autopsies or pedagogical cadaveric dissection.

## Introduction

Normally, each side of the body has a single, non-bifurcating ureter that runs from one ureteropelvic junction along the retroperitoneum, across the psoas muscle and iliac vessels, and along the lateral pelvic wall to a single ureterovesical junction [1]. On the other hand, each side of the body normally has only one testicular artery (TA) that originates inferior to the renal vessels and courses anterior to the IVC without arching over any part of the renal pedicle [2].

Embryologically, the urinary and genital systems are interrelated as they develop from the urogenital ridge in the fourth week of gestation. Part of the urogenital ridge called the gonadal ridge, forms the reproductive system, while another part, known as the nephrogenic cord, eventually becomes the urinary tract. The kidneys form from a part of the nephrogenic cord called the metanephros and begin filtering as early as week eleven of gestation [3]. The mesonephros generates the ureteric bud, which can abnormally bifurcate and lead to an incomplete double ureter. In contrast, two ureteric buds can cause a complete double ureter [1]. Duplex ureter is the most common congenital variation of the urinary tract. Incomplete duplication, which presents as a Y-shape, has a prevalence of 0.6% while complete duplication has a prevalence of 0.2% [4]. 

Rostral to the metanephros, the mesonephros proliferates on its ventromedial surface to form the genital ridge, which gives rise to the testes [5]. Each mesonephros contains a mesonephric duct (Wolffian duct) and a paramesonephric duct (Mullerian duct), which respectively contribute to male and female reproductive structures. The male reproductive structures include the epididymis, seminal vesicles, and vas deferens. During weeks five and six of gestation, primordial germ cells colonize the genital ridge and determine male or female sex [3]. Testes first lie in the abdomen near the kidneys, then descend and eventually pass the inguinal rings to the scrotum after week twenty-one of gestation [6]. Each TA usually originates from the caudal group of lateral mesonephric arteries. However, in some cases, the TA may arise from more cephalic mesonephric arteries [7]. Typically, one right and one left TA arise from the abdominal aorta (AA) inferior to the renal artery (Balci’s type 4 origin). Alternatively, the TA may arise from the AA above the normal level (Balci's type 1 and 2), from a renal, accessory renal, segmental renal, or middle suprarenal (Balci’s type 3). Besides variations in the origin of the TA, variations in its course have also been reported. The TA most commonly courses inferolateral along the posterior abdominal wall to reach the corresponding testis (Balci’s type a). However, the right TA may pass posterior to the Inferior Vena Cava in a retrocaval course (Balci’s type b), or arch around a renal vein (Balci’s type c) [2]. It is not clear why retrocaval or arching TAs occur. Paraskevas cites Notkovitch, whose work is unavailable, to suggest the kidney ascends much higher, carrying the ipsilateral renal vein with it. This ascent is much higher than the level of the TA origin, and as a result, the artery is forced to arch over that vein [8]. Moreover, one could argue that an atypical rotation of the kidneys before they pass the testicles could cause TAs to arch over renal veins, passing anterior to the kidneys instead of behind them, as is more common. 

This case report presents a unique combination of congenital ureteric and testicular arterial anomalies. By documenting the combination of these anomalies, this report emphasizes the importance of in-depth knowledge of urogenital anomalies and contributes to the limited body of scientific literature on possible associations between these anomalies. 

## Case presentation

During a routine laboratory dissection assignment of the posterior abdominal wall at William Carey University College of Osteopathic Medicine, multiple anomalies of the urogenital system were identified, dissected, and examined in a 94-year-old male cadaveric donor. The donor was received from the University of Southern Alabama Anatomical Gift Program. 

**Figure 1:**
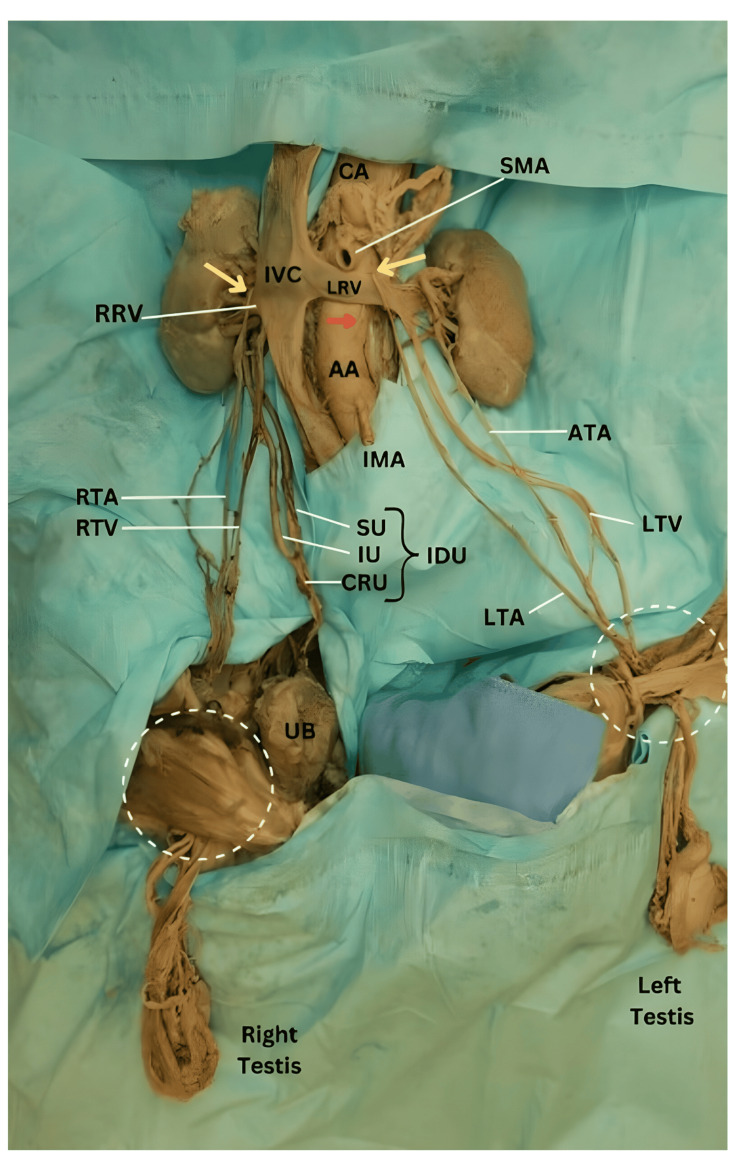
Overview of urogenital structures White dotted circles: the inguinal canal regions; red arrow: origin of the main left testicular artery; yellow arrows: arching testicular arteries of Luschka; AA: abdominal aorta; ATA: accessory testicular artery; CA: celiac artery, CRU: common right ureter; IDU: incomplete double ureter; IMA: inferior mesenteric artery; IU: inferior ureter; IVC: inferior vena cava; LRV: left renal vein; LTA: left testicular artery; LTV: left testicular vein; RRV: right renal vein; RTA: right testicular artery; RTV: right testicular vein; SMA: superior mesenteric artery; SU: superior ureter

**Figure 2:**
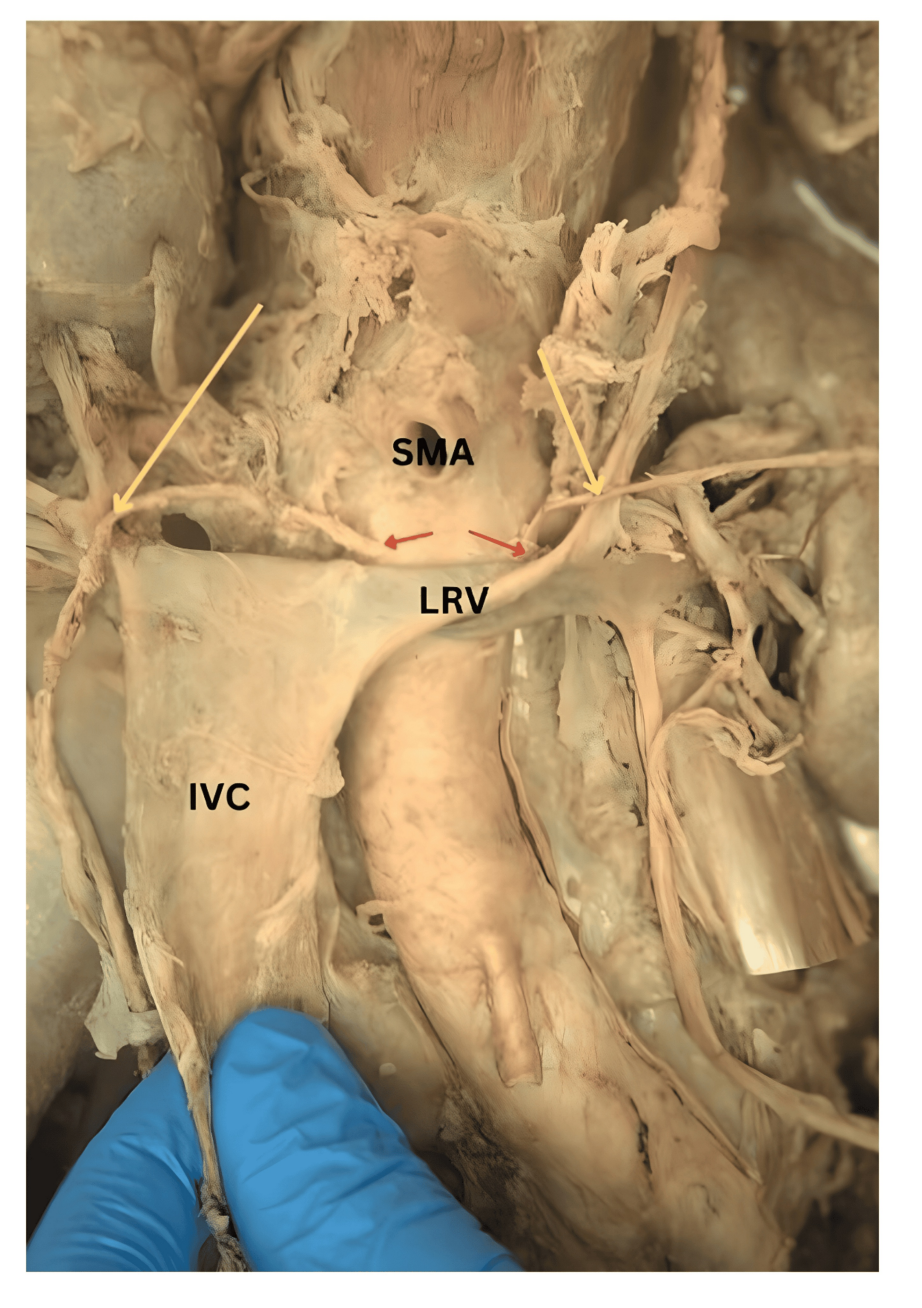
The anterior view of the posterior abdominal wall with IVC reflected inferiorly to show the origin and the retrocaval course of the RTA and the origin of the LTA deep to LRV Red arrows: origin of testicular arteries; yellow arrows: arching testicular arteries; IVC: inferior vena cava; LRV: left renal vein; SMA: superior mesenteric artery; LTA: left testicular artery; RTA: right testicular artery

**Figure 3:**
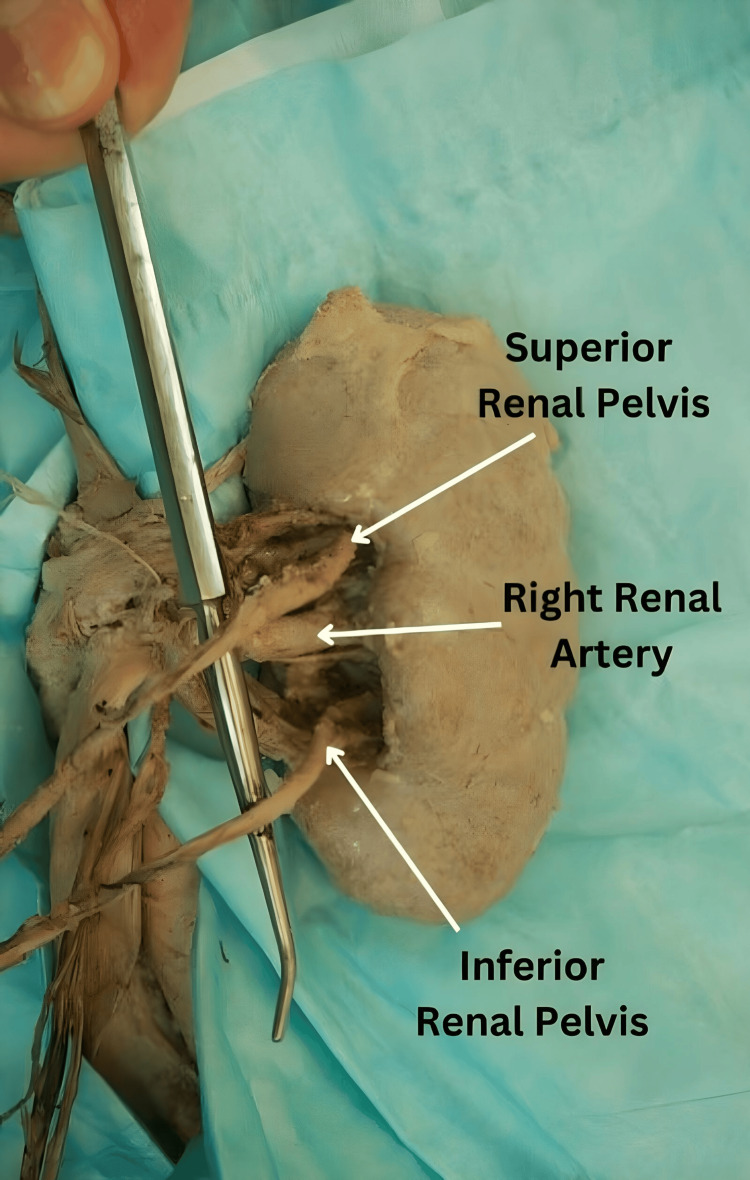
Right kidney drained by duplex renal pelvis into two ureters, kidney reflected medially

Using an anterior approach, the gastrointestinal tract and organs were removed from the abdominal cavity to expose the posterior abdominal wall and viscera. As the peritoneum was stripped off to expose the posterior abdominal wall and viscera, a double TA was observed on the left side, and arching of TAs was noticed bilaterally (Figure 1 and Figure 2). Moreover, the right kidney was drained by an incomplete double ureter (Figure 1 and Figure 3).

**Figure 4:**
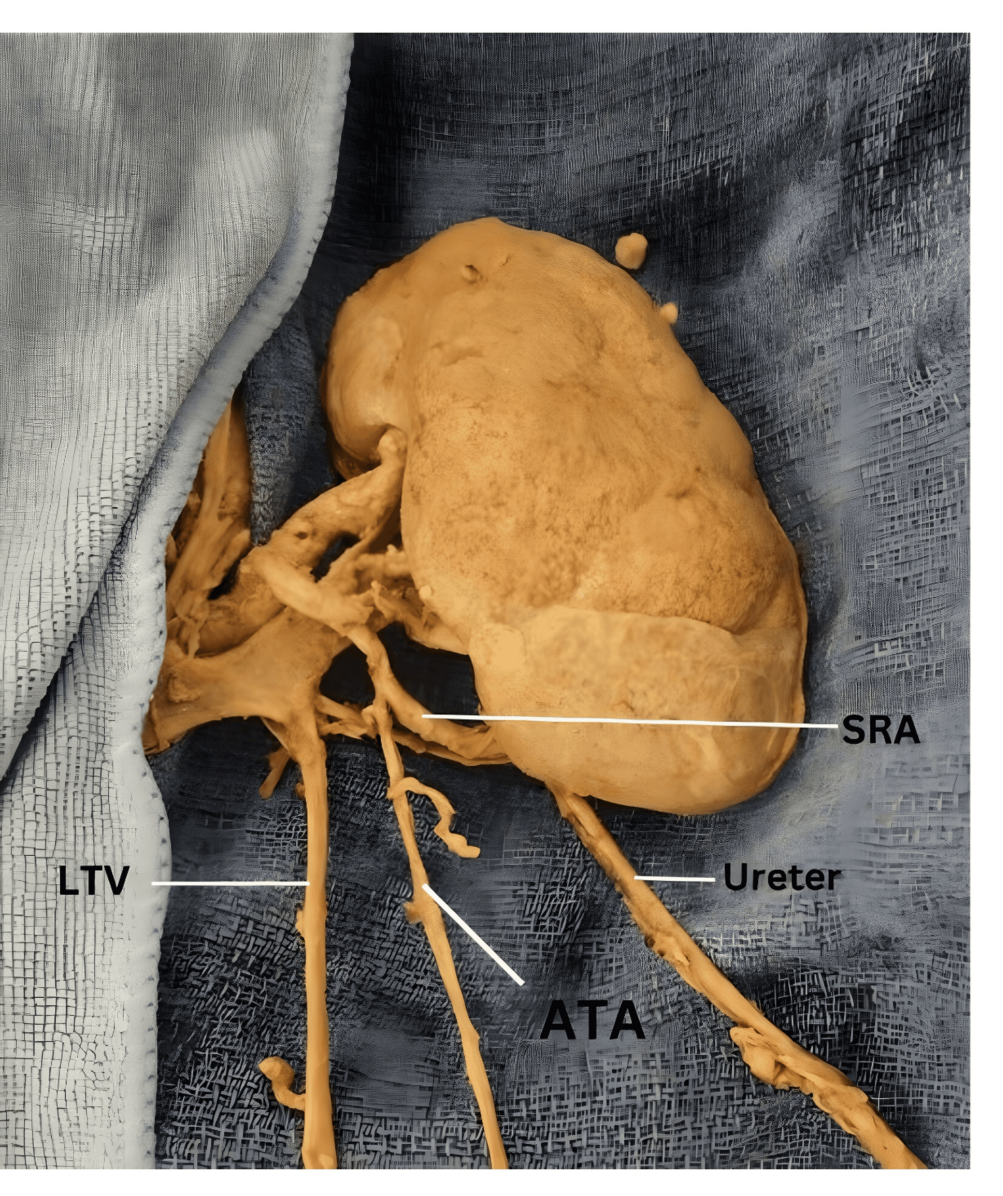
Left kidney showing origin of the ATA ATA: accessory testicular artery; LTV: left testicular vein; SRA: segmental renal artery.

On the left side, there were two TAs originating from two different parent vessels at the level of the body of L3. The main left TA emerged from the AA, 2.6 cm inferior to superior mesenteric artery (SMA), and 0.8 cm lateral to the centerline of AA (Figure 2). It ascended superolaterally for 2.3 cm deep to the left renal vein (LRV) and then looped anteriorly around the LRV to descend inferiorly toward the left testis. The entire length of the main left TA was 36.7 cm. The left accessory testicular artery (ATA) originated from the ipsilateral inferior segmental renal artery and descended for 30.8 cm to reach the left testis (Figure 1 and Figure 4). On the other hand, the right testicular artery arose from the AA at the level of L2-L3 intervertebral space, 1.4 cm inferior to the SMA, and 0.6 cm lateral to the centerline of AA (Figure 2). It ran in a superolateral direction for 3.2 cm, deep to the IVC, and then looped anteriorly around the right renal vein as it joined the IVC. From its origin to its termination in the testis, the length of the right TA measured 37.2 cm (Figure 1). 

Dissection of the hila of the kidneys revealed a right incomplete duplex ureter (Figure 1 and Figure 3). The two ureters originated from two separate renal pelvises and descended for some distance, and then joined together to form a common ureter (a Y-shaped configuration) anterior to the right psoas major muscle. The common ureter descended over the right psoas major and the right common iliac artery and terminated in the superolateral angle of the urinary bladder. The superior ureter arising from the superior renal pelvis ran for 14.2 cm, while the inferior ureter originating from the inferior renal pelvis ran for 12.8 cm, at which point the two joined. The length of the right common ureter (the stem of the Y) measured 9.1 cm.

## Discussion

Congenital anomalies of the kidney and urinary tract (CAKUT) present in a spectrum of severity. The presentation ranges from asymptomatic to numerous urological and nephrological comorbidities. CAKUT, including double ureter, constitutes approximately 30% of all prenatally identified anomalies [9]. Double ureters are categorized as either complete or incomplete in duplication. Incomplete duplication is more common and has two ureters that leave the kidney and then join before reaching the bladder, forming the shape of a “Y” [3]. The most common complications of an incomplete double ureter are ureteroureteral reflux and ureteropelvic junction obstruction [10]. Early detection, therefore, is crucial to prevent these complications. In the present case, we observed an incomplete, Y-shaped, double ureter. However, the sizes of the pelvises and ureter were found to not be dilated, indicating no reflux or obstruction. 

The presence of an incomplete double ureter increases the likelihood of ureteral injury during surgery and associated secondary complications [11]. One report claims that complete double ureters are prone to severe, complex forms of secondary obstructing ureteric or vesical lithiasis, UTIs, or chronic urothelial inflammation [12]. Good imaging must be coupled with thorough knowledge of CAKUT variations to diagnose possible associated complications. Reporting these cases would contribute to literature helping categorize ureter anomalies and the potential complications associated with these anomalies. 

The TAs in this case report are classified according to Balci's system as type 4bc for the right, type 4c for the left main, and type 3a for the left accessory [7]. The duplication and arched course of TAs may lead to cases of varicocele, hydronephrosis, nephroptosis, and renal infarction [13]. An accessory renal artery has been reported alongside bilateral micro-kidneys [14]. The presence of a left ATA originating from the inferior segmental artery increases the risk of arterial injury and hemorrhage during operations on or near the kidneys. Moreover, an arched TA may compress the renal vein [15]. This phenomenon should be considered as a possible etiology in the workup of nutcracker syndrome. We suggest that if a patient presents with the symptoms of pelvic congestion syndrome, hematuria, or varicocele without compression by the SMA, clinicians should consider the possibility of an arching TA variant compressing the renal vein. Even though the TA has a smaller diameter and is more compliant than the SMA, the arching course of the vessel and its downward traction may have a “strangulating” effect on the renal vein. 

Coincidence of variations in the present case may indicate a possible developmental association between these anomalies. While the association between double ureters and anomalous bilateral arching testicular arteries and unilateral accessory testicular arteries has not been reported, coincidences of double ureters and renal artery variations have been documented [16,17]. Several studies have investigated the crucial role of Foxd1 signaling in kidney patterning from the renal stroma and elucidating early embryonic development [18]. Additionally, research highlights the significant contribution of the stroma to the development of various components such as the branching ureteric bud, nephron, tubules, cortex, medulla, and notably, kidney vasculature. Stromal cells differentiate into pericytes and smooth muscle cells, playing a pivotal role in patterning the vascular network of the kidney [19]. The variations in the present case might be due to the closely related embryological origins and patterning of the urinary and genital systems. Further investigation is needed to elucidate the incidence, embryological basis, and clinical significance of the coincidence of these variations. 

Since both arching testicular arteries and double ureters may result in urological symptoms, coexistence of these variations, as seen in this case, may result in confusion in explaining and treating the actual cause of the patient’s urological symptoms as the physician would focus on and manage the more common condition, the double ureter. Utilizing the appropriate radiologic modality is invaluable to correctly identify the presence of duplex ureter, and TA, and renal artery variants. Multi-detector computed tomography (MDCT) and contrast-enhanced MR are excellent tools for simultaneously assessing not only renal blood vessels and parenchyma but also the collecting system and surrounding structures [20]. However, Rubin et al. claim that MR alone is inadequate to assess renal vasculature before surgery [21]. MDCT technology is a non-invasive method for evaluating structures such as testicular arteries [8]. CT angiography is a fast and efficient means of assessing renal vasculature [22]. 

## Conclusions

This case shows a possible association between an incomplete double ureter, arching testicular arteries and an accessory testicular artery. To our knowledge, this report is the first to identify and describe the coexistence of these anomalies and their possible clinical implications. Both double ureter and these arterial anomalies can cause urological symptoms and therefore must be distinguished. Furthermore, arching testicular arteries may cause nutcracker syndrome and should be especially considered as a possible cause when work-up reveals no compression by the SMA. This possible etiology of nutcracker syndrome has been suggested in at least several articles but has not been studied in depth or proven to exist. This case report highlights the possible association between three examples of CAKUT and therefore, unlike other articles, proposes that one look twice after finding an anomaly in order to detect other variations and correctly identify the root cause of the patient’s symptoms. Our report contributes to the limited literature and highlights the importance of reporting these anomalies when encountered during autopsies or pedagogical cadaveric dissection. A comprehensive understanding and identification of variations in testicular vessels and renal structures, along with considering potentially associated complications, are essential for accurate diagnosis and safe surgical interventions.
